# *Leishmania *amastigotes as targets for drug screening

**DOI:** 10.1186/1475-9292-5-6

**Published:** 2006-10-23

**Authors:** Adriano Monte-Alegre, Ali Ouaissi, Denis Sereno

**Affiliations:** 1 IRD, UR008 " Pathogènie des Trypanosomatidés", 911 Avenue Agropolis, BP 64501, 34394 Montpellier Cedex 5, France

## Abstract

Direct drug screening against the mammalian stage of *Leishmania *has been hampered by cost and the time consuming effort required to accomplish it. The ability to derive transgenic *Leishmania *expressing reporter genes opened up new possibilities for the development of drug screening tests. Further developments to standardize and gather multiple informations could now be envisionned. We will discuss on such available methodologies that could improve sensitivity, reliability, versatility and the rapidity, of the screen based on intracellular model.

## Background

*Leishmania *is a protozoan parasite that is responsible for several pathologies collectively known as leishmaniasis. According to the latest WHO, 12 million people are affected by leishmaniasis worldwide and 2 million new cases occur each year [1]. Moreover, the of rise Leishmaniasis is due to multiple factors including the AIDS epidemic, increase of international travel, a lack of effective vaccines, difficulties in controlling vectors, international conflicts and the development of resistance to chemotherapy. Methodologies that closely mimic the conditions encountered by *Leishmania *are required. In this focus, we discuss the potential application of the reporter gene technology, in multiplexing experiments, as a future strategy for drug screening against intracellular *Leishmania *amastigotes.

## Reporter gene technology for drug screening against intracellular amastigotes

The term reporter gene is used to define a gene with a readily measurable phenotype that can be distinguished easily over a background of endogenous proteins [2]. Various recombinant parasites carrying a reporter gene either as an episomal copy or after its integration in a defined locus, generally the rDNA locus, is currently available. For some of them, the capacity of these cell lines to be used for *in vitro *drug screening procedure was evaluated against axenic or intramacrophagic amastigotes (See table 1). Autofluorescent proteins present the advantage of no requirement in cofactors or substrates since the protein is intrinsically fluorescent [3]. Various *Leishmania *species expressing GFP, multimeric GFP or enhanced GFP (eGFP) were engineered [4-10]. Generally transfectants do not express sufficient levels of fluorescence for spectrofluorometric measurement on microplate. To overcome this kind of problem a multimeric form of the GFP was engineered and expressed in *Leishmania *promastigotes. As expected, parasites expressing the multimeric GFP form bear fluorescence quantifiable in 96 wells with spectrofluorometric analysis [6].

**Table 1:** *Leishmania *expressing reporter gene whose capacity to be used *in vitro *for drug screening procedure has been determined

Species	Reporter gene	Expression	Ref
*L. donovani*/*L. donovaniR*	Firefly luciferase	Episomal	16
*L. amazonensis*	Firefly luciferase	Integration	17
*L. infantum*/*L. infantum RSbIII*^1^	Firefly luciferase	Episomal	14
*L. donovani*	GFP	Episomal	7
*L. donovani R*^2^			10
*L. amazonensis*	β-Lactamase	Episomal	12
*L. major*			

^1 ^*in vitro *selected trivalent antimony resistant *L. infantum*. ^2 ^Field isolates of *L. donovani *resistant to pentavalent antimony.

Generally, methods that use catalytic reporter genes technology like luciferase, β-galactosidase, β-lactamase are more sensitive than methods based on fluorescent proteins. Promastigotes of *Leishmania *expressing β-galactosidase were selected and their use in drug screening procedures evaluated [8]. β-galactosidase presents the advantage that colorimetric detection can be performed. However some commonly cited drawbacks of β-galactosidase include its large size (the monomer is 116 kDa) and the endogenous expression of β-galactosidase by some mammalian cell types including macrophages [11-13]. To circumvent these shortcomings, a catalytic reporter system based on β-lactamase was developed [13]. Two species of *Leishmania*: *Leishmania major *and *Leishmania amazonensis *expressing β-lactamase were engineered and selected. Activity of some standard antileishmanial drugs was evaluated on intramacrophagic amastigotes. Overall, the results obtained demonstrate that this methodology could be valuable for drug screening procedures [12].

Various species of parasites expressing luciferase were recently developed and their susceptibility towards classical antileishmanial agents investigated [14-16]. The main advantages of this technology are numerous and include the high sensitivity of the test and the absence of background activity in the host cell. Recently, a refined work performed by Lang and co-workers demonstrated that *L. amazonensis *parasites expressing firefly luciferase could be used to monitor *Leishmania *infection in real time, through imaging analysis. They have also tested various antileishmanial compounds and have followed their efficacy in live cells by using imaging [17]. The advantage of this methodology rely on the capacity to perform experiments on live cells, making the analysis faster and more accurate since viability of both the parasites and the host cells is monitored.

Reporter genes present several limitations. Cross resistance conferred by the presence of the antibiotic resistance is one of them. Neomycin confers resistance toward paromomycin [18]. The development of method to create defined mutants lacking selectable markers could help to overcome this problem [19]. The way by which the reporter gene is introduced could also have an impact on the throughput of the screen. When reporters are part of plasmids, the relative output of reporter may depend on the copy number of the transfected plasmid (which vary from cell to cell) rather than on the activity of the drug. Secondly transforming parasites could have biological consequences either by disrupting the genomic architecture or just by the presence of the foreign reporter gene product. Thirdly, for the β-galactosidase technology, the reporter could have by itself some limitations (*i.e *sensibility, background activity from host macrophages) that make it inaccurate for an *in vitro *determination of drug activity against intracellular parasites.

## Improving standardization and the effectiveness of drug screening by gathering multiple informations simultaneously

Getting information on drug activity against an intracellular organism is difficult and time consuming. The toxicity data against the host cell must be gathered before testing the compound against the intracellular pathogen. Heterogeneity in the infection rate of the host may have an influence on the compound toxicity. The kinetic activity of the compounds may vary and have to be evaluated. A versatile methodology allowing the investigator to quantify and test multiple parameters of kinetic of action, drug concentration and viability against both the host cells and the intracellular amastigotes (multiplexing) could increase the throughput of the screen. To simultaneously gather information on the viability of the host cells and the parasites the use of a combination of parasites and macrophages expressing different reporter can now be envisioned. To achieve this goal, reporter must use distinguishable signal from each other and use compatible chemistries. Fluorophores, that emit different wavelengths, have been widely used to distinguish among multiple signals. Recently, there have been a growing number of examples using luminescence for multiplexing either in combination with: 1- other luminescent signals, 2- fluorescence or 3-β-galactosidase assay [20,21]. Since the results are expressed as a ratio between the output signal emitted by the host cell and the one emitted by parasites, such methodology could also help to standardize the experiments in order to directly compare drug activity. The usefulness of these approaches for drug screening has to be evaluated on intracellular parasites like *Leishmania *or *T. cruzi*. Fibroblast expressing β-galactosidase can be purchased at the ATCC and may thus represent good candidates to perform preliminary experiments, on *T. cruzi*. In conclusion, the capacity of multiple gene reporter technologies to be used in multiplexing experiments have to be evaluated since they may represent valuable tools in the field of parasitology and pharmacology.

## References

[B1] WHO. http://www.who.int/leishmaniasis/en/.

[B2] Naylor LH (1999). Reporter gene technology: the future look bright. Biochem Pharmacol.

[B3] Tsien RY (1998). The green fluorescent protein. Annu Rev Biochem.

[B4] Ha DS, Schwarz JK, Turco SJ, Beverley SM (1996). Use of the green fluorescent protein as a marker in transfected *Leishmania*. Mol Biochem Parasitol.

[B5] Misslitz A, Mottram JC, Overath P, Aebischer T (2000). Targeted integration into a rRNA locus results in uniform and high level expression of transgenes in *Leishmania *amastigotes. Mol Biochem Parasitol.

[B6] Chan MMY, Bulinski JC, Chang KP, Fong DA (2003). Microplate assay for *Leishmania amazonensis *promastigotes expressing multimeric green fluorescent protein. Parasitol Res.

[B7] Singh N, Dube A (2004). Fluorescent *Leishmania*: aplication to anti-leishmanial drug testing. Am J Tro Med Hyg.

[B8] Okuno T, Goto Y, Matsumoto Y, Otsuka H, Matsumoto Y (2003). Applications of recombinant *Leishmania amazonensis *expressing egfp or the beta-galactosidase gene for drug screening and histopathological analysis. Exp Anim.

[B9] Kamau SW, Grimm F, Hehl AB (2001). Expression of green flrorescent protein as a marker for effect of antileishmanial compounds *in vitro*. Antimicrobial Agents Chemother.

[B10] Dube A, Singh N, Sundar S, Singh N (2005). Refractoriness to the treatment of sodium stibogluconate in Indian kala-azar field isolates persist in *in vitro *and *in vivo *experimental models. Parasitol Res.

[B11] Campbell RE (2005). Realization of β-lactamase as a versatile fluorogenic reporter. Trends in Biotechnol.

[B12] Buckner FS, Wilson AJ (2005). Colorimetric assay for screening compounds against *Leishmania *amastigotes grown in macrophages. Am J Trop Med Hyg.

[B13] Zlokarnik G, Negulescu PA, Knapp TE, Mere L, Burres N, Feng L, Whitney M, Roemer K, Tsien RY (1998). Quantitation of transcription and clonal selection of single living cells with beta-lactamase as reporter. Science.

[B14] Sereno D, Roy G, Lemesre JL, Papadopoulou B, Ouellette M (2001). DNA transformation of *Leishmania infantum *axenic amastigotes and their use in drug screening. Antimicrob Agents Chemother.

[B15] Roy G, Dumas C, Sereno D, Wu Y, Singh AK, Tremblay MJ, Ouellette M, Olivier M, Papadopoulou B (2000). Episomal and stable expression of the luciferase reporter gene for quantifying *Leishmania spp*. infections in macrophages and in animal models. Mol Biochem Parasitol.

[B16] Gupta AS, Ramesh Sundar S, Goyal N (2005). Use of *Leishmania donovani *field isolates expressing the luciferase reporter gene in *in vitro *drug screening. Antimicrob Agents Chemother.

[B17] Lang T, Goyard S, Lebastard M, Milon G (2005). Bioluminescent *Leishmania *expressing luciferase for rapid and high throughput screening of drugs acting on amastigote-harbouring macrophages and for quantitative real-time monitoring of parasitism features in living mice. Cell Microbiol.

[B18] Kutzleb R, Schweyen RJ, Kaudewitz F (1973). Extrachromosomal inheritance of paromomycin resistance in *Saccharomyces cerevisiae*. Molecular Genetics and Genomics.

[B19] Denise H, Coombs GH, Mottram JC (2004). Generation of *Leishmania *mutants lacking antibiotic resistance genes using a versatile hit-and-run targeting strategy. FEMS Microbiol Lett.

[B20] Young KH, Wang Y, Bender C, Ajit S, Ramirez F, Gilbert A, Nieuwenhuijsen BW (2004). Yeast-based screening for inhibitors of RGS proteins. Methods Enzymol.

[B21] Grover GS, Turner BA, Parker CN, Meier J, Lala DS, Lee PH (2003). Multiplexing nuclear receptors for agonist identification in a cell-based reporter gene high-throughput screen. J Biomol Screen.

